# Supplementary material for: Evaluation of the diagnostic accuracy of two point-of-care tests for COVID-19 when used in symptomatic patients in community settings in the UK primary care COVID diagnostic accuracy platform trial (RAPTOR-C19)
Source: PLoS One. 2023 Jul 21;18(7):e0288612. doi: 10.1371/journal.pone.0288612 (PMC10361479; doi:10.1371/journal.pone.0288612)
Supplement: S1 File — (DOCX) [file pone.0288612.s001.docx]

Supplemental Material S1

# Further details of index testing procedures

Consumables for SD Biosensor tests came from three manufacturing LOTs, while those for BD Veritor tests came from a single manufacturing LOT. Four SD Biosensor tests were drawn at random from separate kits of 25 and were tested using SD Biosensor Standard COVID-19 Ag Positive and Negative control materials (SD Biosensor Inc, Gyeonggi-do, Republic of Korea) to check test LOT integrity ahead of deployment to study sites. This process was repeated for each new LOT deployed. The integrity of each BD Veritor kit of 30 assays was checked by site staff using SARS-CoV-2 positive and negative control swabs provided by the manufacturer as a component of each kit.

The SD Biosensor Standard™ Q SARS-CoV-2 Rapid Antigen Test required nasopharyngeal swabs to be taken from participants. Swabs were immediately extracted in buffer tubes through a combination of stirring and squeezing, with a minimum of 5 swab rotations required. Three drops of eluate were dispensed into the specimen well of the assay through buffer tube dispenser caps. Test results were visually interpreted following ≥15 to ≤30 minutes incubation. Results were deemed invalid if a test control line was not visible following incubation. Bilateral nares swab samples were collected from participants for the BD Veritor™ assay. Swabs were immediately extracted for a minimum of 15 seconds by plunging up and down in buffer tube fluid. Processed swabs were withdrawn from buffer tubes whilst squeezing the tube sides to extract the majority of liquid. Tubes were subsequently capped and 3 drops of eluate dispensed into the assay sample well using the integrated dropper cap. Test cartridges were read after a 15 minute incubation period using the BD Veritor™ Plus Analyzer.

# Summary of interim analysis for SD Biosensor

An interim analysis was performed for the SD Biosensor POCT, using all data available up to 30th July 2021, to determine whether early termination for futility should be recommended. Early termination would occur only if both the sensitivity and the specificity of the point-of-care test lay below their respective futility thresholds. These futility thresholds were conservatively set as 75% for sensitivity and 90% for specificity. Adapting the group-sequential test for a proportion in a Fleming clinical trial design, with a one-sided type I error rate of 2.5% and a type II error rate of 20%, an interim analysis performed after 98 of the target number of 150 test-positive samples had been obtained resulted in futility boundaries of 27 false negative results and 26 false positive results.

At this date, 331 participants had available results for both the SD Biosensor test and the RT-PCR reference test. Of the 97 participants with a positive reference test result, 80 were correctly identified as positive by the SD Biosensor test and there were 17 false negative results (estimated sensitivity 82.5%). Of 234 participants with a negative reference test result, 231 were correctly identified as negative and there were 3 false positive results (estimated specificity 98.7%).

As neither the number of false negative nor the number of false positive results exceeded the calculated futility boundaries, there was insufficient evidence to recommend termination futility for either sensitivity or specificity at the interim analysis stage. It was further noted that the estimated sensitivity and specificity values at the interim stage both lay between the ‘acceptable’ and ‘desired’ levels specified by the MHRA Target Product Profile (sensitivity: 80%=acceptable, 95%=desired; specificity: 95%=acceptable, 99%=desired).

**S1 Table 1: Diagnostic performance by subgroup.**

| **Index test** | **Subgroup** | **TP** | **FN** | **FP** | **TN** | **Sensitivity** | **Specificity** | **PPV** | **NPV** |
| --- | --- | --- | --- | --- | --- | --- | --- | --- | --- |
|  | All participants | 178 | 34 | 5 | 328 | 0.84 (0.78, 0.89) | 0.99 (0.97, 1.00) | 0.97 (0.94, 0.99) | 0.91 (0.87, 0.93) |
|  | Male | 100 | 11 | 2 | 116 | 0.90 (0.83, 0.95) | 0.98 (0.94, 1.00) | 0.98 (0.93, 1.00) | 0.91 (0.85, 0.96) |
|  | Female | 78 | 23 | 3 | 212 | 0.77 (0.68, 0.85) | 0.99 (0.96, 1.00) | 0.96 (0.90, 0.99) | 0.90 (0.86, 0.94) |
|  | Adults (age 16+ years) | 174 | 32 | 3 | 298 | 0.84 (0.79, 0.89) | 0.99 (0.97, 1.00) | 0.98 (0.95, 1.00) | 0.90 (0.87, 0.93) |
|  | Age < 16 years | 4 | 2 | 2 | 30 | 0.67 (0.22, 0.96) | 0.94 (0.79, 0.99) | 0.67 (0.22, 0.96) | 0.94 (0.79, 0.99) |
| SD Biosensor | Age 16-39 years | 70 | 17 | 1 | 128 | 0.80 (0.71, 0.88) | 0.99 (0.96, 1.00) | 0.99 (0.92, 1.00) | 0.88 (0.82, 0.93) |
|  | Age 40-59 years | 77 | 11 | 1 | 102 | 0.88 (0.79, 0.94) | 0.99 (0.95, 1.00) | 0.99 (0.93, 1.00) | 0.90 (0.83, 0.95) |
|  | Age 60+ years | 27 | 4 | 1 | 68 | 0.87 (0.70, 0.96) | 0.99 (0.92, 1.00) | 0.96 (0.82, 1.00) | 0.94 (0.86, 0.98) |
|  | White ethnicity | 155 | 31 | 4 | 278 | 0.83 (0.77, 0.88) | 0.99 (0.96, 1.00) | 0.97 (0.94, 0.99) | 0.90 (0.86, 0.93) |
|  | Non-white ethnicity | 23 | 3 | 1 | 50 | 0.88 (0.70, 0.98) | 0.98 (0.90, 1.00) | 0.96 (0.79, 1.00) | 0.94 (0.84, 0.99) |
|  | Main symptoms – none | 30 | 8 | 1 | 83 | 0.79 (0.63, 0.90) | 0.99 (0.94, 1.00) | 0.97 (0.83, 1.00) | 0.91 (0.83, 0.96) |
|  | Main symptoms – one | 37 | 14 | 3 | 161 | 0.73 (0.58, 0.84) | 0.98 (0.95, 1.00) | 0.93 (0.80, 0.98) | 0.92 (0.87, 0.96) |
|  | Main symptoms – two or more | 111 | 12 | 1 | 84 | 0.90 (0.84, 0.95) | 0.99 (0.94, 1.00) | 0.99 (0.95, 1.00) | 0.88 (0.79, 0.93) |
|  | Recruited from general practice | 129 | 24 | 5 | 243 | 0.84 (0.78, 0.90) | 0.98 (0.95, 0.99) | 0.96 (0.92, 0.99) | 0.91 (0.87, 0.94) |
|  | Recruited from testing centre | 49 | 10 | 0 | 85 | 0.83 (0.71, 0.92) | 1.00 (0.96, 1.00) | 1.00 (0.93, 1.00) | 0.89 (0.81, 0.95) |
|  | All participants | 127 | 39 | 3 | 249 | 0.77 (0.69, 0.83) | 0.99 (0.97, 1.00) | 0.98 (0.93, 1.00) | 0.86 (0.82, 0.90) |
|  | Male | 65 | 15 | 1 | 80 | 0.81 (0.71, 0.89) | 0.99 (0.93, 1.00) | 0.98 (0.92, 1.00) | 0.84 (0.75, 0.91) |
|  | Female | 62 | 24 | 2 | 169 | 0.72 (0.61, 0.81) | 0.99 (0.96, 1.00) | 0.97 (0.89, 1.00) | 0.88 (0.82, 0.92) |
|  | Adults (age 16+ years) | 122 | 38 | 3 | 225 | 0.76 (0.69, 0.83) | 0.99 (0.96, 1.00) | 0.98 (0.93, 1.00) | 0.86 (0.81, 0.90) |
|  | Age < 16 years | 5 | 1 | 0 | 24 | 0.83 (0.36, 1.00) | 1.00 (0.86, 1.00) | 1.00 (0.48, 1.00) | 0.96 (0.80, 1.00) |
| BD Veritor | Age 16-39 years | 46 | 14 | 1 | 97 | 0.77 (0.64, 0.87) | 0.99 (0.94, 1.00) | 0.98 (0.89, 1.00) | 0.87 (0.80, 0.93) |
|  | Age 40-59 years | 51 | 17 | 2 | 80 | 0.75 (0.63, 0.85) | 0.98 (0.91, 1.00) | 0.96 (0.87, 1.00) | 0.82 (0.73, 0.89) |
|  | Age 60+ years | 25 | 7 | 0 | 48 | 0.78 (0.60, 0.91) | 1.00 (0.93, 1.00) | 1.00 (0.86, 1.00) | 0.87 (0.76, 0.95) |
|  | White ethnicity | 116 | 36 | 3 | 234 | 0.76 (0.69, 0.83) | 0.99 (0.96, 1.00) | 0.97 (0.93, 0.99) | 0.87 (0.82, 0.90) |
|  | Non-white ethnicity | 11 | 3 | 0 | 15 | 0.79 (0.49, 0.95) | 1.00 (0.78, 1.00) | 1.00 (0.72, 1.00) | 0.83 (0.59, 0.96) |
|  | Main symptoms – none | 21 | 10 | 0 | 51 | 0.68 (0.49, 0.83) | 1.00 (0.93, 1.00) | 1.00 (0.84, 1.00) | 0.84 (0.72, 0.92) |
|  | Main symptoms – one | 27 | 14 | 3 | 120 | 0.66 (0.49, 0.80) | 0.98 (0.93, 0.99) | 0.90 (0.73, 0.98) | 0.90 (0.83, 0.94) |
|  | Main symptoms – two or more | 79 | 15 | 0 | 78 | 0.84 (0.75, 0.91) | 1.00 (0.95, 1.00) | 1.00 (0.95, 1.00) | 0.84 (0.75, 0.91) |
|  | Recruited from general practice | 65 | 20 | 2 | 119 | 0.76 (0.66, 0.85) | 0.98 (0.94, 1.00) | 0.97 (0.90, 1.00) | 0.86 (0.79, 0.91) |
|  | Recruited from testing centre | 62 | 19 | 1 | 130 | 0.77 (0.66, 0.85) | 0.99 (0.96, 1.00) | 0.98 (0.91, 1.00) | 0.87 (0.81, 0.92) |

TP: true positive, FN: false negative, FP: false positive, TN: true negative, PPV: positive predictive value, NPV: negative predictive value. 95% confidence intervals in brackets. Participants were classified as exhibiting main symptoms if they had one, or more than one, of the following symptoms: fever, cough, change in sense of taste, or loss in sense of smell.


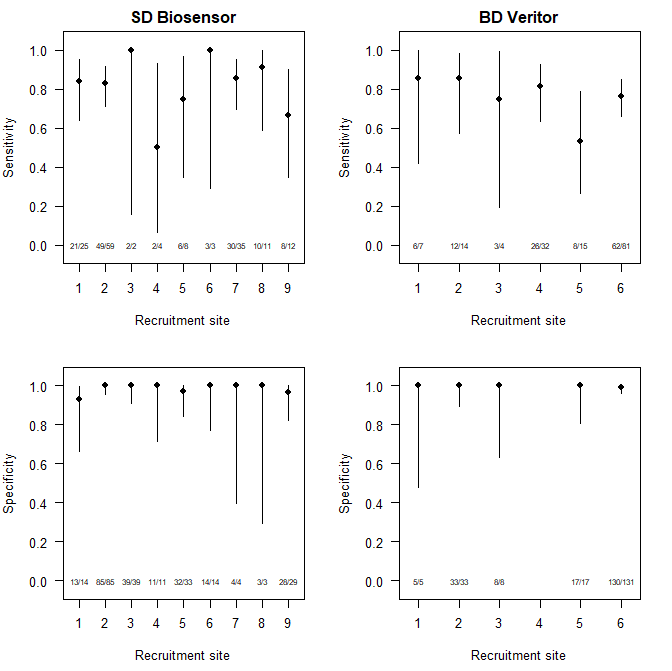


**S1 Figure 1. Estimated sensitivity (upper two panels) and specificity (lower two panels), with 95% confidence intervals, by recruitment site, for SD Biosensor (left two panels) and BD Veritor (right two panels).** The number of individuals correctly diagnosed by the POCT out of the total are shown towards the bottom of each plot. Sites are arbitrarily numbered 1-9 for SD Biosensor graphs and 1-6 (not necessarily the same sites) for BD Veritor graphs. Only sites that contributed at least 10 participants for the primary analysis are shown. For BD Veritor, site 4 did not recruit any participants who received a negative RT-PCR results.


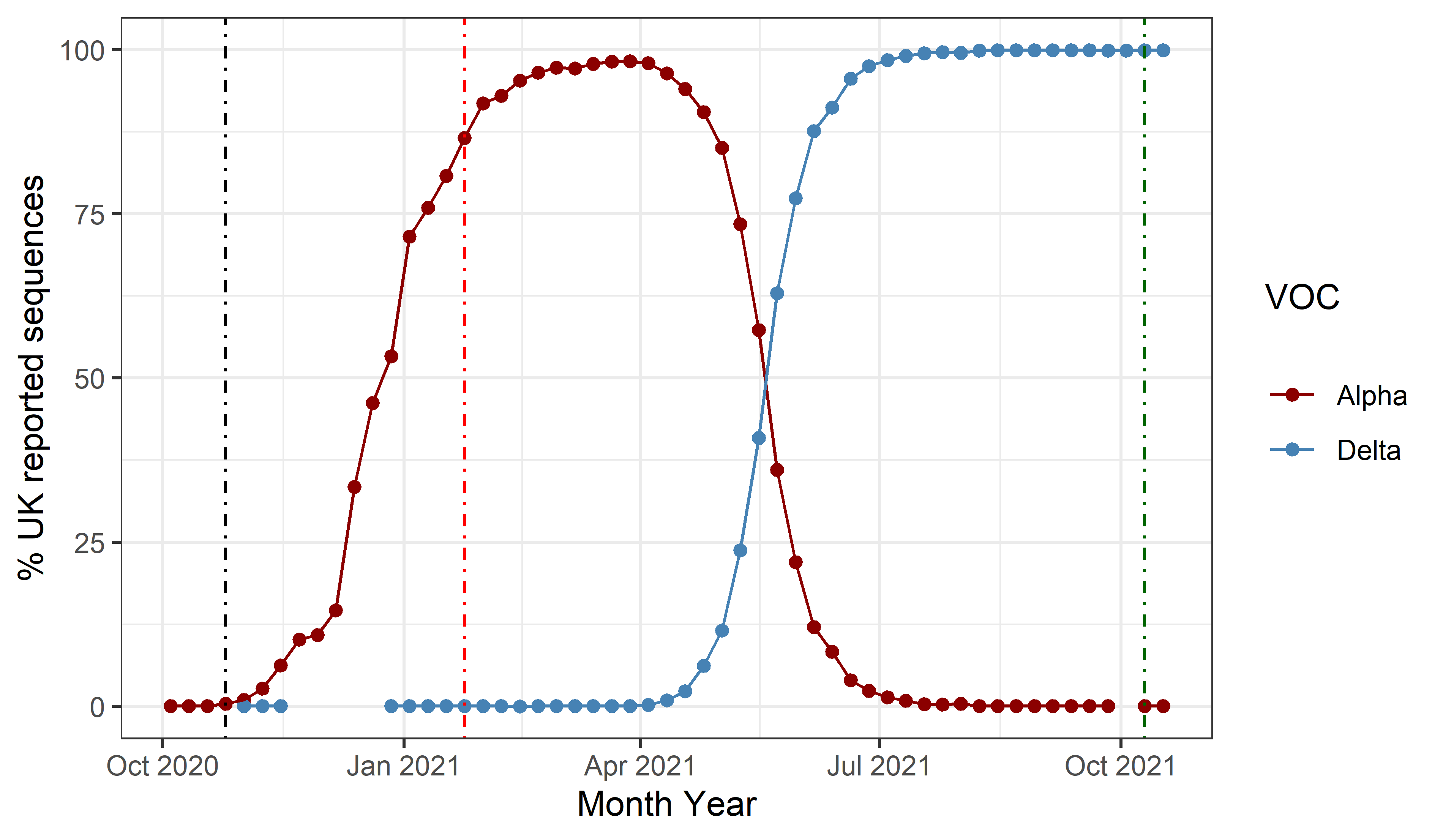


**S1 Figure 2. Relative SARS-CoV-2 variant sequence frequency for the UK during the study course, for the dominant VOC Alpha GRY (B.1.1.7+Q.) and VOC Delta GK (B.1.617.2+AY.) variants.** Vertical dotted lines plotted to the nearest data point represent the start of recruitment for the SD Biosensor assay (black), start of recruitment for the BD Veritor assay (red) and the end of recruitment for both assays (dark green). Source data were downloaded from Gisaid on 14/06/2022.


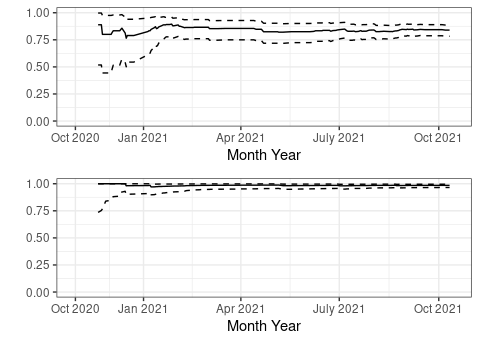


**S1 Figure 3. Estimated sensitivity (top panel) and specificity (bottom panel) of SD Biosensor, with 95% confidence intervals (dotted lines), over time.**


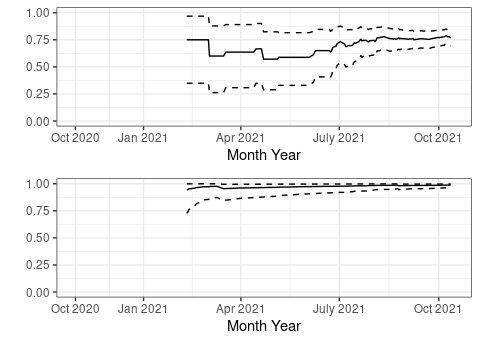


**S1 Figure 4. Estimated sensitivity (top panel) and specificity (bottom panel) of BD Veritor, with 95% confidence intervals (dotted lines), over time.**

**S1 Table 2: Summary of diagnostic performance in relation to RT-PCR cycle threshold, by sex.**

| **MALES** |  | ORF1ab | | | | E gene | | | |
| --- | --- | --- | --- | --- | --- | --- | --- | --- | --- |
|  | Ct value | ≤ 20 | 20-25 | 25-30 | ≥ 30 | ≤ 20 | 20-25 | 25-30 | ≥ 30 |
| SD Biosensor | Positive | 30 | 49 | 17 | 4 | 36 | 43 | 18 | 3 |
|  | Negative | 0 | 1 | 4 | 5 | 0 | 1 | 4 | 6 |
|  | Sensitivity | 1.00 (0.88, 1.00) | 0.98 (0.89, 1.00) | 0.81 (0.58, 0.95) | 0.44 (0.14, 0.79) | 1.00 (0.90, 1.00) | 0.98 (0.88, 1.00) | 0.82 (0.60, 0.95) | 0.33 (0.07, 0.70) |
| BD Veritor | Positive | 24 | 33 | 6 | 1 | 30 | 27 | 6 | 2 |
|  | Negative | 0 | 3 | 5 | 7 | 0 | 3 | 6 | 6 |
|  | Sensitivity | 1.00 (0.86, 1.00) | 0.92 (0.78, 0.98) | 0.55 (0.23, 0.83) | 0.12 (0.00, 0.53) | 1.00 (0.88, 1.00) | 0.90 (0.73, 0.98) | 0.50 (0.21, 0.79) | 0.25 (0.03, 0.65) |
| **FEMALES** |  | ORF1ab | | | | E gene | | | |
|  | Ct value | ≤ 20 | 20-25 | 25-30 | ≥ 30 | ≤ 20 | 20-25 | 25-30 | ≥ 30 |
| SD Biosensor | Positive | 28 | 32 | 16 | 2 | 32 | 29 | 15 | 2 |
|  | Negative | 0 | 5 | 7 | 10 | 0 | 5 | 8 | 10 |
|  | Sensitivity | 1.00 (0.88, 1.00) | 0.86 (0.71, 0.95) | 0.70 (0.47, 0.87) | 0.17 (0.02, 0.48) | 1.00 (0.89, 1.00) | 0.85 (0.69, 0.95) | 0.65 (0.43, 0.84) | 0.17 (0.02, 0.48) |
| BD Veritor | Positive | 35 | 23 | 4 | 0 | 39 | 19 | 4 | 0 |
|  | Negative | 1 | 3 | 12 | 7 | 1 | 3 | 12 | 8 |
|  | Sensitivity | 0.97 (0.85, 1.00) | 0.88 (0.70, 0.98) | 0.25 (0.07, 0.52) | 0.00 (0.00, 0.41) | 0.97 (0.87, 1.00) | 0.86 (0.65, 0.97) | 0.35 (0.07, 0.52) | 0.00 (0.00, 0.37) |


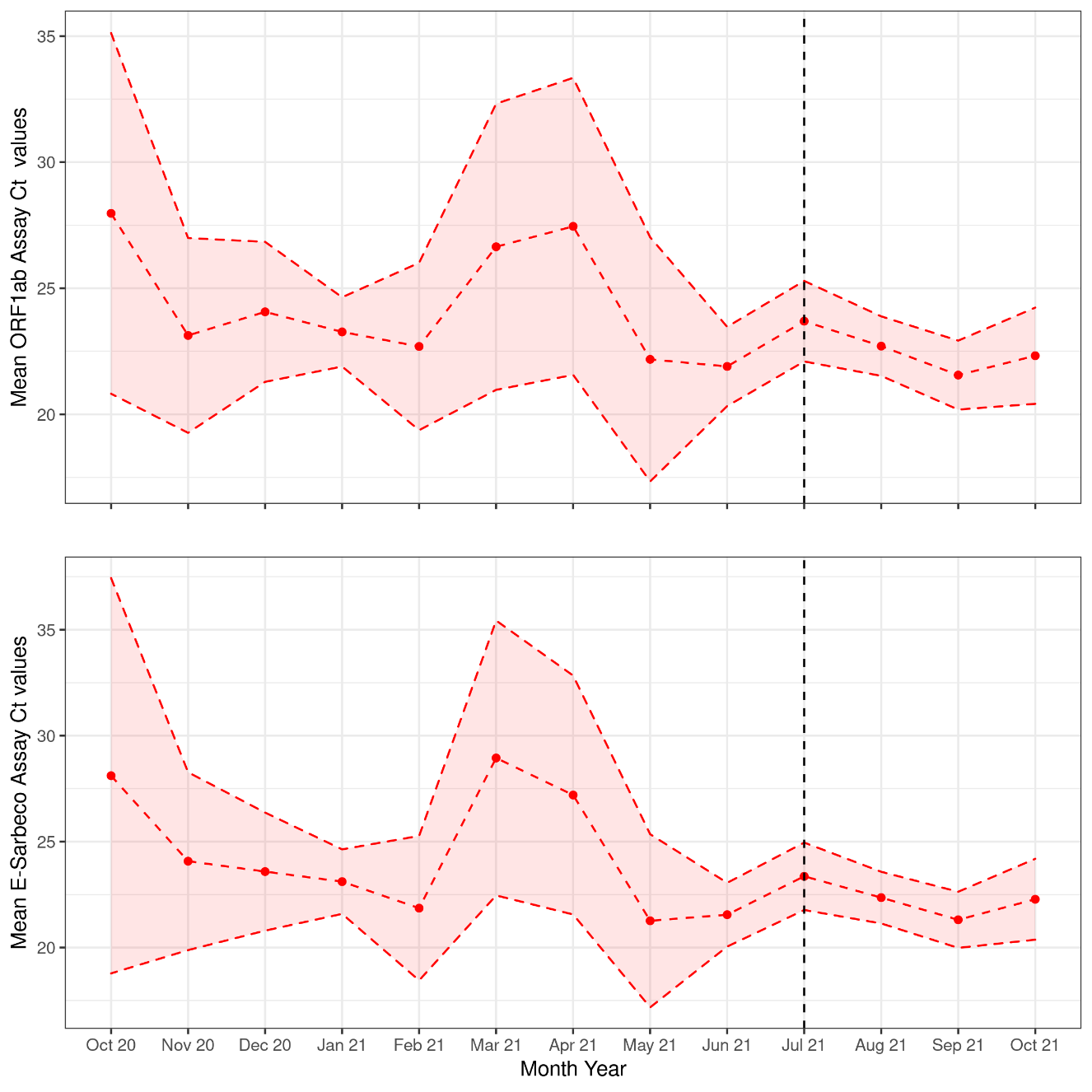


**S1 Figure 5. Mean monthly trend in Ct values (and 95% confidence intervals) for ORF1ab gene assay target (upper panel) and E. Sarbeco gene assay target (lower panel).** The time point at which the testing centre started recruitment is indicated by the dotted black line.

# Summary of enhanced reference standard results

**S1 Table 3. Summary of findings using enhanced reference standard, for participants with discordant index test and RT-PCR results.**

| **Test results** | | | **Further information for enhanced reference standard** | **Indicative result of enhanced reference standard** | **N** | **Total N** |
| --- | --- | --- | --- | --- | --- | --- |
| **SD Biosensor** | **BD Veritor** | **Reference standard (RT-PCR)** |  |  |  |  |
| Negative | Not performed | Positive | Additional positive RT-PCR and positive follow-up serology | Supports positive result | 7 | 13 |
|  |  |  | Additional positive RT-PCR, no follow-up serology | Supports positive result | 3 |  |
|  |  |  | No additional information | No evidence | 2 |  |
|  |  |  | Negative serology at 20-day follow-up | Does not support positive result | 1 |  |
| Positive | Not performed | Negative | Negative baseline and follow-up serology | Supports negative result | 1 | 4 |
|  |  |  | Alternative diagnosis: respiratory syncytial virus | Supports negative result | 1 |  |
|  |  |  | No additional information | No evidence | 2 |  |
| Not performed | Negative | Positive | Additional positive RT-PCR, no follow-up serology | Supports positive result | 3 | 10 |
|  |  |  | Baseline serology positive in vaccinated participant, no follow-up serology | Limited evidence | 1 |  |
|  |  |  | No additional information | No evidence | 6 |  |
| Not performed | Positive | Negative | Spike protein negative at baseline and positive at 28 days in vaccinated participant | Limited evidence | 1 | 1 |
| Negative | Negative | Positive | Additional positive RT-PCR and positive follow-up serology | Supports positive result | 3 | 17 |
|  |  |  | Additional positive RT-PCR and positive baseline serology 11 days after symptom onset, no follow-up serology | Supports positive result | 1 |  |
|  |  |  | Additional positive RT-PCR, no follow-up serology | Supports positive result | 6 |  |
|  |  |  | Negative baseline and positive follow-up serology | Supports positive result | 1 |  |
|  |  |  | No additional information | No evidence | 6 |  |
| Negative | Positive | Negative | No additional information | No evidence | 1 | 2 |
|  |  |  | Spike protein negative at baseline and positive at 28 days in unvaccinated participant | Does not support negative result | 1 |  |
| Negative | Positive | Positive | No additional information | No evidence | 4 | 4 |
| Positive | Negative | Negative | No additional information | No evidence | 1 | 1 |
| Positive | Negative | Positive | Participant hospitalised for COVID-19, additional positive RT-PCR and positive follow-up serology | Supports positive result | 1 | 12 |
|  |  |  | Additional positive RT-PCR and positive follow-up serology | Supports positive result | 2 |  |
|  |  |  | Additional positive RT-PCR, no follow-up serology | Supports positive result | 2 |  |
|  |  |  | No additional information | No evidence | 7 |  |

An enhanced reference standard was created by considering, in combination, antibody testing (serology) results at baseline and 28-day follow-up (nucleocapsid protein and spike protein, recorded here as positive/negative for could/could not be detected, respectively, by the UKHSA method); reported RT-PCR test results that may have been conducted additional to the swab used for the main reference standard (for example, if the participant sought a subsequent confirmatory RT-PCR test); and linked hospitalisation and mortality records. Not all of these supplementary pieces of information were available for all participants.

Results from the 64 participants who had a discordant result between either or both index tests (Columns 1 & 2) and the reference standard (Column 3) are summarised in the table above. Column 4 (‘Further information for enhanced reference standard’) outlines the supplementary information used to inform a decision about the enhanced reference standard. Column 5 (‘Indicative result of enhanced reference standard’) indicates whether the balance of this supplementary information tends to support (highlighted green), or not support (highlighted red), the primary reference standard result (Column 3). Decisions were taken upon discussion between two co-authors.

In only two cases was there judged to be a sufficient degree of evidence to indicate possible reference standard misclassification. The implications are that one SD Biosensor false negative result would be reclassified as a true negative, although this would be offset by one SD Biosensor true negative result being reclassified as a false negative. One BD Veritor false positive result would be reclassified as a true positive.

# Summary of imperfect reference standard statistical adjustment

To assess the extent of possible imperfect reference standard bias, we adjusted to sensitivity and specificity estimates using a Bayesian adjustment approach.

Beta prior distributions for the sensitivity and specificity of the reference standard were derived from performance characteristics of the RT-PCR test in operation during the study period with the following assumptions:

- Prior for reference standard sensitivity followed a Beta distribution with mean 97%, and 95% of the density lying inside the interval [95%, 99%], corresponding to a Beta(α=252.5, β=7.778) distribution.
- Prior for reference standard specificity followed a Beta distribution with mean 99%, and 95% of the density lying inside the interval [98%, 100%], corresponding to a Beta(α=345.3, β=3.478) distribution.
- Uninformative Beta(α=1, β=1) distributions (equivalent to Uniform[0,1]) for each index test specificity and specificity, and disease prevalence.

Posterior distributions for the sensitivity and specificity of both index tests are shown in S1 Fig 6 and summarised in S1 Table 4.


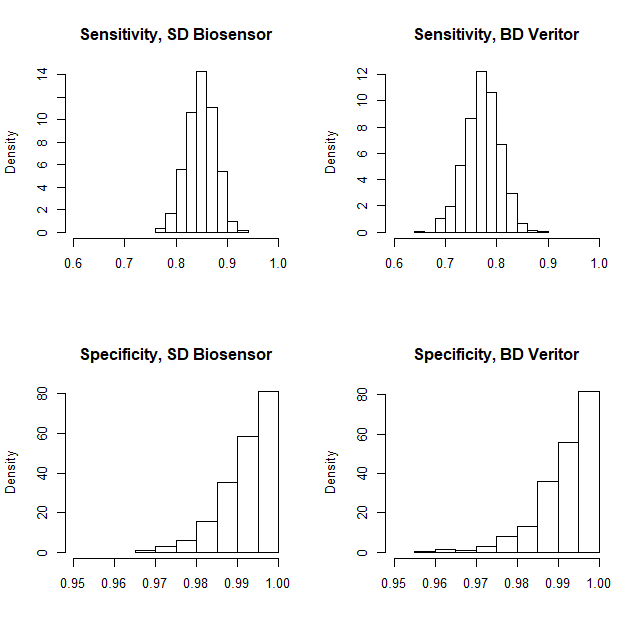


**S1 Figure 6. Posterior distributions for sensitivity and specificity of index tests.**

**S1 Table 4. Posterior medians (central 95% credible intervals) of posterior distributions for sensitivity and specificity of index tests, compared with estimates (95% confidence intervals) from primary analysis.**

| Index test | Measure | Estimate (95% confidence interval), from unadjusted primary analysis | Posterior median (95% credible interval) after imperfect reference standard adjustment |
| --- | --- | --- | --- |
| SD Biosensor | Sensitivity | 0.840 (0.783, 0.886) | 0.850 (0.795, 0.868) |
|  | Specificity | 0.985 (0.965, 0.995) | 0.994 (0.977, 0.997) |
| BD Veritor | Sensitivity | 0.765 (0.693, 0.827) | 0.774 (0.752, 0.795) |
|  | Specificity | 0.988 (0.966, 0.998) | 0.994 (0.975, 0.997) |

**S1 Table 5: Diagnostic performance sensitivity analyses.**

| **Index test** | **Subgroup** | **TP** | **FN** | **FP** | **TN** | **Sensitivity** | **Specificity** | **PPV** | **NPV** |
| --- | --- | --- | --- | --- | --- | --- | --- | --- | --- |
|  | Primary analysis | 178 | 34 | 5 | 328 | 0.84 (0.78, 0.89) | 0.99 (0.97, 1.00) | 0.97 (0.94, 0.99) | 0.91 (0.87, 0.93) |
| SD Biosensor | More strict scenario | 144 | 24 | 5 | 289 | 0.86 (0.79, 0.91) | 0.98 (0.96, 0.99) | 0.97 (0.92, 0.99) | 0.92 (0.89, 0.95) |
|  | Less strict scenario | 178 | 35 | 5 | 346 | 0.84 (0.78, 0.88) | 0.99 (0.97, 1.00) | 0.97 (0.94, 0.99) | 0.91 (0.87, 0.94) |
|  | Primary analysis | 127 | 39 | 3 | 249 | 0.77 (0.69, 0.83) | 0.99 (0.97, 1.00) | 0.98 (0.93, 1.00) | 0.86 (0.82, 0.90) |
| BD Veritor | More strict scenario | 110 | 32 | 3 | 239 | 0.77 (0.80, 0.84) | 0.99 (0.96, 1.00) | 0.97 (0.92, 0.99) | 0.88 (0.84, 0.92) |
|  | Less strict scenario | 127 | 39 | 3 | 250 | 0.77 (0.69, 0.83) | 0.99 (0.97, 1.00) | 0.98 (0.93, 1.00) | 0.86 (0.82, 0.90) |

TP: true positive, FN: false negative, FP: false positive, TN: true negative, PPV: positive predictive value, NPV: negative predictive value. 95% confidence intervals in brackets.

‘More strict scenario’ corresponds to excluding all individuals whose recruitment date did not exactly match the recorded swab date.

‘Less strict scenario’ corresponds to excluding all individuals whose recorded swab date did not fall within 7 days of the recruitment date.
