# Supplementary material for: Evaluation of the diagnostic accuracy of two point-of-care tests for COVID-19 when used in symptomatic patients in community settings in the UK primary care COVID diagnostic accuracy platform trial (RAPTOR-C19)
Source: PLoS One. 2023 Jul 21;18(7):e0288612. doi: 10.1371/journal.pone.0288612 (PMC10361479; doi:10.1371/journal.pone.0288612)
Supplement: S2 File — (DOCX) [file pone.0288612.s002.docx]

STATISTICAL ANALYSIS PLAN

RAPTOR-C19

**Final version**

**Date: 22/11/2021**

Version History

| Version: | Version Date: | Changes: |
| --- | --- | --- |
| 1.0 | 30/11/2020 | Original version |
| 1.1 | 23/7/2021 | Updated Sections 3.4 and 5: interim analysis for first point-of-care test |
| 1.2 | 30/7/2021 | Section 4.4: add subgroup analysis for recruitment via testing centres |
| 1.3 | 6/9/2021 | Minor correction to Section 2.2 |
| 1.4 | 8/11/2021 | Add cross-classification of multiple index tests as secondary outcome; add comparison of index test result and PCR cycle threshold; amend baseline characteristics |
| 2.1 | 15/11/2021 | Add information about batch/lot numbers of index tests. Version finalised ahead of data analysis. |
| 2.2 | 22/11/2021 | Amend Appendix 2 to match protocol. Other minor typographical corrections. |

# Table of Contents

[Table of Contents 2](#_Toc72144478)

[1 Introduction 3](#_Toc72144479)

[1.1 Purpose and scope of the plan 3](#_Toc72144480)

[1.2 Trial overview 3](#_Toc72144481)

[1.3 Objectives 3](#_Toc72144482)

[2 Study design 4](#_Toc72144483)

[2.1 Outcome measures 4](#_Toc72144484)

[2.1.1 Primary outcome 4](#_Toc72144485)

[2.1.2 Secondary outcomes 4](#_Toc72144486)

[2.2 Sample size 4](#_Toc72144487)

[3 Analysis – General considerations 6](#_Toc72144488)

[3.1 Characteristics of participants 6](#_Toc72144489)

[3.2 Eligibility of participants 6](#_Toc72144490)

[3.3 Pooling of investigational sites 6](#_Toc72144491)

[3.4 Interim analyses 6](#_Toc72144492)

[3.5 Reporting 7](#_Toc72144493)

[4 Data analysis 7](#_Toc72144494)

[4.1 Primary outcome (using laboratory reference standard) 7](#_Toc72144495)

[4.2 Secondary outcome (using enhanced laboratory reference standard) 7](#_Toc72144496)

[4.3 Handling missing data 8](#_Toc72144497)

[4.4 Subgroup analyses 8](#_Toc72144498)

[4.5 Adverse events 9](#_Toc72144499)

[5 Changes to the protocol or previous versions of sap 9](#_Toc72144500)

[6 References 10](#_Toc72144501)

[7 Appendices 11](#_Toc72144502)

[7.1 Appendix 1 – Sample size table 11](#_Toc72144503)

[7.2 Appendix 2 – Outline of enhanced reference standard 11](#_Toc72144504)

#

# Introduction

## Purpose and scope of the plan

This statistical analysis plan (SAP) should be read alongside the study protocol (Nicholson 2021), which gives a detailed explanation of the rationale of the study and the study design.

This version of the SAP outlines the analytic procedure for the evaluation of diagnostic point-of-care tests (POCTs) in which no interim or futility analysis is planned. An updated version of the SAP will apply to evaluations of POCTs that require an interim analysis, informed by the initial complete evaluations.

Different POCTs will be evaluated separately and may require adaptations of the basic design to allow for sufficient evaluation, depending on their intended use and position in the diagnosis and treatment pathway. As required, deviations from this overarching SAP will be described in appendices to meet the requirements of the POCT under assessment.

## Trial overview

RAPTOR-C19 is a platform study to evaluate multiple POCTs, including those triaged for community evaluation by the COVID-19 National DiagnOstic Research and Evaluation Platform (CONDOR).

The aim of RAPTOR-C19 is to assess the diagnostic accuracy of multiple current and emerging POCTs for active or past COVID-19 infection in the community setting.

## Objectives

Primary:

- Assess the standard diagnostic accuracy of POCTs for active COVID-19 infection compared to the Public Health England (PHE) reference laboratory standard or equivalent.

Secondary:

- Assess the standard diagnostic accuracy of POCTs for past COVID-19 infection compared to the PHE laboratory reference standard.
- Assess the diagnostic accuracy of POCTs for active COVID-19 infection against an enhanced composite reference standard using multiple tests data, linked Electronic Health Records (EHR) data, and patient reported outcomes data.
- Assess the diagnostic accuracy of POCTs for past COVID-19 infection against an enhanced composite reference standard using multiple tests data, linked EHRs, and patient reported outcomes data.

# Study design

RAPTOR-C19 incorporates a series of prospective observational parallel diagnostic accuracy studies of COVID-19 POCTs against laboratory and composite reference tests in patients with suspected current or past COVID-19 attending community settings such as general practice.

Patients contributing data to this study will typically undergo the POCT (‘index test’) under consideration and a laboratory reference standard test consisting of both antigen and serology tests. The current PHE reference standard for active infection is an oropharyngeal/nasopharyngeal swab (SARS-Cov-2 d/r RT-PCR) and the reference standard for past infection is serology for laboratory antibody testing.

Participants will have a baseline assessment that records demographic and symptom information. Adults (≥ 16 years) will additionally complete a 28-day follow up visit for additional serology (antibody test) alongside a symptom diary (Nicholson 2021, Table 1).

Both adults and children presenting at general practice with suspected COVID-19 infection, or having a swab for laboratory COVID-19 testing, are eligible for participation (Nicholson 2021, Box 1).

## Outcome measures

### Primary outcome

- Sensitivity, specificity, and positive and negative predictive values for each index test in relation to the laboratory reference test. These diagnostic measures refer to either active or past infection, depending on the nature of the POCT under evaluation.

### Secondary outcomes

- Sensitivity, specificity, and positive and negative predictive values for the index test in relation to the composite reference standard. These diagnostic measures refer to either active or past infection, depending on the nature of the POCT under evaluation.
- Cross-classification of results of each index test, in relation to the laboratory reference standard, among samples in which more than one index test was performed.
- The number of missing test results for the index test and for the reference standard against which it is being compared.

## Sample size

Sample size considerations described here are indicative only and may vary depending on the evaluation requirements of individual POCTs. In addition to the statistical issues described in this section, the decision about the appropriate sample size for evaluating any particular POCT will depend on additional factors including the number of tests available to the investigatory team, and the availability of practices from which study participants are recruited. Any factors that materially affect the choice of sample size for a particular POCT will be justified in an appendix.

A table of illustrative sample sizes for sensitivity, based on a standard error of 2.5% and corresponding to an approximate confidence interval width of ±5%, was included in Nicholson 2021 and is reproduced here for convenience in Section 7.1. For sample size based on the sensitivity, the determining factor is the number of individuals who are reference standard-positive. For example in the case that the true sensitivity of the index test is 90%, a total of 144 reference standard-positive individuals would be required in the scenario outlined in Appendix 1. For a prevalence of COVID-19 of 10%, this would correspond to a total sample size of 1440.

In a formal test of the sensitivity (a test for a single proportion), if the true sensitivity of the index test were 90%, a total of 108 reference standard-positive patients would have 80% power to detect a difference from a minimum requirement of 80% sensitivity, and a total of 137 reference standard-positive patients would have 90% power to detect the same effect.

These numbers are broadly consistent with the POCT Target Product Profile provided by the Medicines & Healthcare products Regulatory Agency (MHRA), which recommends at least 150 positive clinical samples and designates sensitivity of greater than 80% as “acceptable” and sensitivity of greater than 97% as “desired” (MHRA 2020). A similar sample size (n=156 or more reference standard-positive samples) would be sufficient to exclude sensitivity parameters of 97% or below (based on the lower limit of the 95% confidence interval) if, and only if, all samples were correctly classified as positive by the index test. This figure therefore represents a target sample size for meeting the “desired” sensitivity level specified by the MHRA for a test that shows perfect performance in the current study.

As expected prevalence levels are expected to be substantially lower than 50%, the number of reference standard-negative samples is likely to be much higher than the number of reference standard-positive samples. MHRA recommendations for specificity are for greater than 95% as “acceptable” and greater than 99% as “desired” (MHRA 2020). Consequently, the precision of estimates of specificity will be much higher than that for sensitivity, and confidence intervals will be narrower.

As an example, in the same scenario as described above (a total sample size of 1440, with 10% prevalence of COVID-19), a true specificity of 99% would be expected to be estimated with 95% confidence interval limits of 98.2% to 99.4%. The lower limit of an exact 95% confidence interval for the specificity would exclude the “desired” level of 99% if n=476 reference standard-negative samples were all correctly classified as negative by the index test. This sample size would be comfortably attained while meeting sample size requirements for the sensitivity, provided the prevalence of COVID-19 is less than around 25%, as would be expected.

Of note, the set of COVID-19 diagnostic test requirements published on the website of FIND specifies desired sensitivity and specificity levels of greater than 99% for a diagnostic test that will be used for definitive diagnosis without confirmatory testing (FIND 2020). Given the sample size considerations outlined above, it is unlikely that this requirement would be definitively met by an evaluation in the current study unless it achieves correct diagnostic results on all samples, for which the issue of an imperfect reference standard may become important (Section 4.2).

# Analysis – General considerations

## Characteristics of participants

The demographic variables listed below will be collected at baseline and summarised as frequencies and percentages, or as mean and standard deviation, or median and interquartile range, as appropriate for the data type, and with appropriate tables and graphs. Summaries will be presented separately for adults (≥ 16 years old) and children (< 16 years old).

Age
Gender
Ethnicity
Household contacts
Care home resident
Past COVID test results
Vaccinated against COVID

Summaries of the variables listed below, relating to the baseline symptoms of the participants and the timing of symptoms (whether currently present, and start and end dates, as appropriate), will also be presented.

Well-being (1-10 scale)

Recovery from symptoms (binary)

Others in household with respiratory illness (binary)

Specific symptoms (on scale of 1=No problem to 4=Major problem), including but not limited to the following: Fever, Cough, Shortness of breath, Muscle ache, Nausea/Vomiting, Other symptoms (to be described by patient)

Swab result, if taken (binary)

Hospital admission, including timing, whether admitted overnight, and stay in Intensive Care Unit

## Eligibility of participants

All participants will be included in the analysis of this study, with the exception of those who have withdrawn voluntarily or been discontinued from the study by the Study Management Committee for reasons including ineligibility or deviation from protocol.

## Pooling of investigational sites

The primary analysis will use data pooled from all sites at which patients were recruited. Between-practice variation will be explored as a subgroup analysis, as described in Section 4.4.

## Interim analyses

The original version of the SAP related to evaluations for which no interim analysis was planned. During the recruitment phase it was decided to undertake an interim analysis of the primary outcome for the first POCT introduced to the study. This interim analysis was based on separate hypothesis tests for sensitivity and specificity, using the exact group sequential method described by Zhao (2007). These hypothesis tests were performed against conservative futility cut-offs of 75% for sensitivity and 90% for specificity, with use of the POCT to terminate if and only if it were judged futile for both sensitivity and specificity. The cut-offs were chosen to be five percentage points lower than those considered ‘acceptable’ by the MHRA (2020), because it was judged desirable to not to terminate use of the POCT in the study unless the evidence to do so was clear-cut, and because at the time of the interim analysis (July 2021), insufficient data for creating an enhanced reference standard were not available. As the interim analysis was intended to assess futility only (i.e. terminating use of the POCT in the event of poor performance), the significance level was adjusted to 0.025 to reflect a one-sided test. The interim analysis for the sensitivity assumed a total target sample size of 150 positive samples (as judged by the laboratory reference standard result). In the interim analysis for the specificity, the expected total number of negative samples was estimated as 150×(1-p)/p, where p is the estimated prevalence of positive samples (as judged by the laboratory reference standard result) at the time of performing the interim analysis.

## Reporting

Reporting will, if appropriate, follow standard guidelines (STARD) for diagnostic studies (Bossuyt 2015).

# Data analysis

## Primary outcome (using laboratory reference standard)

The sensitivity, specificity and positive and negative predictive values for the index test in relation to the laboratory reference test will be presented and proportions with exact 95% confidence intervals, also reporting the numerator and denominator used in the calculations of each, in the form of a 2x2 table. The definition of the primary outcome assumes that the laboratory reference standard provides the correct diagnosis of each sample as positive or negative; the implications of making this assumption are addressed in the analysis of the secondary outcomes (Section 4.2).

Index test results will also be presented in relation to the cycle threshold value for all targets used as part of the reference standard test, both graphically and numerically for cycle threshold categories of <20, <25 and <30. A summary of the number of batches/lots of each index test used will be included.

## Secondary outcome (using enhanced laboratory reference standard)

To address the possibility that the laboratory reference standard does not always provide a correct diagnostic classification, we will create an enhanced composite reference standard and use this as the comparator in calculating summary statistics of diagnostic performance of the index test. Our approach broadly follows methods for dealing with imperfect reference standard bias as described in the overview article by Reitsma (2009). In the presence of an imperfect reference standard, estimates of sensitivity and specificity, such as those calculated in Section 4.1, may be biased (Zhou 2002, Ch. 3.4). In the case of uncorrelated errors in the index test and reference standard, unadjusted estimates will be biased downwards, and for the low range of prevalences likely to be encountered in this study, it is anticipated that the bias in the sensitivity is likely to be larger than that of the specificity (Walter 2012).

This secondary outcome will use an enhanced composite reference standard constructed as a combination of the reference standard test result, patient-reported outcomes, linked electronic health records for outcomes related to COVID-19 such as hospitalisation or death, and other test results, but without use of the index test result to ensure blinding and prevent incorporation bias.

Two approaches to construct such an enhanced reference standard are to attempt to minimise the number of reference standard false negatives, and the other to attempt to minimise the number of reference standard false negatives. A possible approach based on combining laboratory SARS-CoV-2 RNA results (RT-PCR) results with Immunoglobin G (IgG) serology results (if available), subsequent PCR test results and hospitalisation and mortality records was included as Table 3 in Nicholson 2021 and is also presented here in Section 7.2.

The impact of imperfect reference standard bias will also be checked using an assumption that the sensitivity and specificity of the laboratory reference standard are fixed values less than 100%, in the case of an imperfect reference standard (Gart 1966). A range of plausible sensitivity and specificity values will be considered as a sensitivity analysis, using the Bayesian approach to adjustment for imperfect reference standard bias, as described in Zhou (2002, Ch. 11.3) and Lu (2010). Uncertainty in the sensitivity and specificity of the reference test will be captured using Beta prior distributions, with the parameters of these distributions chosen to match the assumed estimate and uncertainty of the reference standard, from previously available information. For example, if the sensitivity estimate of the laboratory reference standard, alongside its 95% confidence interval or credible interval, is available, a Beta prior with parameters that correspond to these estimates is uniquely defined (Wu 2008).

This method will initially assume independence between the index test and reference standard results, although if this analysis suggests that imperfect reference standard bias might substantially affect the interpretation of the diagnostic performance of the reference test, a further sensitivity analysis will be performed that relaxes this assumption by specifying prior distributions for two covariance parameters (the covariances between the index test and reference test result, among those with and without disease respectively). These priors will be specified as continuous Uniform distributions on a domain ranging between zero and the maximum possible upper bound that is consistent with the sensitivities and specificities of the index and reference tests. Methods are described in Vacek (1985), Hui (1998) and Lu (2010).

In the event that more than one POC test is performed on the same sample, a cross-classification of the results of each of these index tests in relation to the laboratory reference standard will be presented. In the case of two index tests, this will produce a 2x2x2 table showing positive and negative results from each of the two index test and from the reference standard.

## Handling missing data

In the primary analysis, results will be presented only for samples that returned results both for the index test and the part of the reference standard appropriate to the index test under consideration (for example, the RT-PCR result if the index test is aimed at detecting active infection). The number of samples for which either the index test result or the reference standard result were missing will be reported, along with the reasons these results were missing, if known, and with a cross-classification against the result of the other test, if this is available. These results will be stratified by children vs adults and by age group, if counts are sufficiently high to make this informative. Potential associations between patterns of missing data and patient characteristics (age, sex and baseline symptoms) will be explored and reported using appropriate tables and graphs.

The number of missing index test results is an important secondary outcome. However, missing data for either the index test or the reference standard can occur for a variety of reasons, and should not necessarily be expected to bias estimates of diagnostic performance. Nevertheless, unexplained missing index test results for which the reference test result is not missing are potentially more concerning than *vice versa*, as they might plausibly reflect borderline or difficult-to-assess samples. For this reason, as a sensitivity analysis, any such samples will be regarded as incorrect diagnostic classifications (false negatives if the reference standard result was positive; false positives if the reference standard result was negative), and estimates of sensitivity and specificity (with 95% confidence intervals) will be recalculated. This will give an indication of a plausible ‘worst case scenario’ that might have eventuated had no missing results for the index test occurred.

## Subgroup analyses

Results for the primary outcome will be stratified by adults vs children, by age group (<16, 16-40, 40-60, 60+ years), by gender, by ethnicity, by spectrum of disease (a combined measure of symptom severity and duration) and by recruitment method (direct recruitment from general practice versus recruitment via testing sites).

A further subgroup analysis will test for differences in diagnostic performance by practice, and if any differences are detected, associations with practice size, location and model of care will be explored.

## Adverse events

Any adverse events judged to be related to study procedures will be reported in the study results, as described by Nicholson (2021).

# Changes to the protocol or previous versions of sap

Any important changes from the statistical analysis section in the protocol or previous versions of the SAP are described here.

1. In version 1.1 of this SAP, Section 3.4 (Interim analysis) was rewritten to describe an interim analysis undertaken for the first POCT introduced into the study.

2. As a result of the change in recruitment strategy to include recruitment from testing centres, a subgroup analysis was added in Section 4.4 to stratify results by recruitment method.

3. The primary outcome analysis section was amended to clarify that index test results would be presented in relation to the PCR cycle threshold.

4. An additional cross-classification of samples in which more than one index test was performed was added as an additional secondary analysis.

# References

Bossuyt PM, et al. STARD 2015: an updated list of essential items for reporting diagnostic accuracy studies. BMJ, 2015. 351:h5527.

FIND. SARS-CoV-2 Diagnostic Use Cases. 2020. Available from: https://www.finddx.org/covid-19/dx-use-cases/.

Gart JJ, Buck AA. Comparison of a screening test and a reference test in epidemiologic studies: II. A probabilistic model for the comparison of diagnostic tests. Am J Epid. 1966; 83(3):593-602.

Hui SL, Zhou XH. Evaluation of diagnostic tests without gold standards. Stat Meth Med Res. 1998; 7:354-70.

Lu Y, Dendukuri N, Schiller I, Joseph L. A Bayesian approach to simultaneously adjusting for verification and reference standard bias in diagnostic test studies. Stat Med. 2010; 29:2532-43.

MHRA (Medicines & Healthcare products Regulatory Agency). Target Product Profile: Point of Care SARS-CoV-2 Detection Tests, v1.0. 2020. Available from: https://assets.publishing.service.gov.uk/government/uploads/system/uploads/attachment_data/file/895745/TPP_Point_of_Care_SARS-CoV-2_Detection_Tests.pdf.

Nicholson BD, et al. Rapid Community Point-of-Care Testing for COVID-19 (RAPTOR-C19): protocol for a platform diagnostic study. Diagn Progn Res. 2021; 5:4.

Reitsma JB, Rutjes AW, Khan KS, Coomarasamy A, Bossuyt PM. A review of solutions for diagnostic accuracy studies with an imperfect or missing reference standard. J Clin Epidemiol. 2009; 62(8):797-806.

Vacek PM. The effect of conditional dependence on the evaluation of diagnostic tests. Biometrics. 1985; 41:959-68.

Walter SD, et al. Effect of dependent errors in the assessment of diagnostic or screening test accuracy when the reference standard is imperfect. Stat Med. 2012; 31:1129-38.

Wu Y, Shih WJ, Moore DF. Elicitation of a Beta prior for Bayesian inference in clinical trials. Biometrical Journal. 2008; 50(2):212-23.

Zhou X, Obuchowski N, McClish D. Statistical Methods in Diagnostic Medicine. 2002. New York: John Wiley & Sons.

Zhao Z. Early stopping clinical trials of binomial response with an exact group sequential method. Stat Med. 2007; 26: 1724-29.

# Appendices

## Appendix 1 – Sample size table

**Illustrative sample sizes to achieve a range of POCT sensitivities based on a standard error of 2.5%.**

|  | **Prevalence** | **40%** | **35%** | **30%** | **25%** | **20%** | **15%** | **10%** | **5%** |
| --- | --- | --- | --- | --- | --- | --- | --- | --- | --- |
| **Sensitivity** | **95%** | 190 | 218 | 254 | 304 | 380 | 507 | 760 | 1520 |
|  | **90%** | 360 | 412 | 480 | 576 | 720 | 960 | 1440 | 2880 |
|  | **85%** | 510 | 583 | 680 | 816 | 1020 | 1360 | 2040 | 4080 |
|  | **80%** | 640 | 732 | 854 | 1024 | 1280 | 1707 | 2560 | 5120 |
|  | **75%** | 750 | 858 | 1000 | 1200 | 1500 | 2000 | 3000 | 6000 |
|  | **70%** | 840 | 960 | 1120 | 1344 | 1680 | 2240 | 3360 | 6720 |

## Appendix 2 – Outline of enhanced reference standard

**Potential use of tests to enhance the reference standard**

|  | **Minimise FN for Current infection** | | **Minimise FN for Past infection** | |
| --- | --- | --- | --- | --- |
| ***Visit (day)*** | *1 (0)* | *2 (28)* | *1 (0)* | *2 (28)* |
| **SARS-CoV-2 RT PCR** | Negative (FN) | N/A | Negative | N/A |
| **COVID IgG** | Negative | Positive | Negative (FN) | Positive |
| *Or:* | Subsequent positive SARS-Cov-2 RT PCR result (within 28 days) | | - | |
| *Or:* | Hospital admission or death (within 28 days) citing SARS-CoV-2 as probable cause | | Hospital admission or death (within 28 days) citing SARS-CoV-2 as probable cause | |
|  | **Minimise FP for Current infection** | | **Minimise FP for Past infection** | |
| ***Visit (day)*** | *1 (day 0)* | *2 (28)* | *1 (day 0)* | *2 (28)* |
| **SARS-CoV-2 RT PCR** | Positive (FP) | N/A | Negative | N/A |
| **COVID IgG** | Negative | Negative | Positive (FP) | Negative |
| *And:* | No subsequent positive SARS-Cov-2 RT PCR result (within 28 days) | | - | |
| *And:* | No hospital admission or death (within 28 days) citing SARS-CoV-2 as probable cause | | No hospital admission or death (within 28 days) citing SARS-CoV-2 as probable cause | |

Information in the table above refers to hypothetical combinations of results in which the enhanced reference standard result would be considered different from the original reference standard result. A cell-by-cell interpretation of each scenario follows.

**Minimise FN (False Negative) for Current infection**: If the COVID-19 RT-PCR reference test gives a negative result, it can be considered a false negative if the IgG result is positive at 28 days (newly positive or titres have increased), OR if a subsequent SARS-Cov-2 RT PCR result (within 28 days) was observed, OR if there was a hospital admission or death (within 28 days) citing SARS-CoV-2 as probable cause.

**Minimise FP (False Positive) for Current infection**: If the COVID-19 RT-PCR reference test gives a positive result, it can be considered a false positive if the IgG result is negative at both baseline and 28 days, AND if no subsequent SARS-Cov-2 RT PCR result (within 28 days) was observed, AND if there was no hospital admission or death (within 28 days) citing SARS-CoV-2 as probable cause.

**Minimise FN (False Negative) for Past infection**: If the baseline reference IgG test result is negative, it can be considered a false negative if the 28-day IgG test result is positive, OR if there was a hospital admission or death (within 28 days) citing SARS-CoV-2 as probable cause.

**Minimise FP (False Positive) for Past infection**: If the baseline reference IgG test result is positive, it can be considered a false positive if all other tests at baseline and at 28 days are negative, AND there has been no hospital admission or death (within 28 days) citing SARS-CoV-2 as probable cause.
